# Unexpected transient femoral nerve palsy following epidural catheterization in total hip arthroplasty: A rare case report

**DOI:** 10.1016/j.ijscr.2025.111651

**Published:** 2025-07-10

**Authors:** Mohammad Poursalehian, Mohammadreza Razzaghof, Pantea Bozorg Savoji, Mohammad Ghorbanzadeh, Moeen Akbari Javar, S.M. Javad Mortazavi

**Affiliations:** a Joint Reconstruction Research Center, Tehran University of Medical Sciences, Tehran, Iran; b Department of Orthopedic Surgery, Imam Khomeini Hospital Complex, Tehran University of Medical Sciences, Tehran, Iran

**Keywords:** Total hip arthroplasty, Spinal anesthesia, Epidural catheter, Case report

## Abstract

**Introduction and importance:**

Total hip arthroplasty is frequently performed under spinal anesthesia, which is generally safe. However, rare neurological complications, such as femoral nerve palsy, may occur. This report describes an unprecedented case of transient femoral nerve palsy following epidural catheterization in total hip arthroplasty.

**Case presentation:**

A 44-year-old male with femoral head avascular necrosis underwent left total hip arthroplasty. An epidural catheter was placed at the L2/3 level for postoperative pain management without complications. Two days postoperatively, the patient developed right-sided femoral nerve palsy. MRI scans revealed no spinal cord compression or hematoma. The patient's neurological symptoms began to improve five days after surgery and fully resolved by the eighth postoperative day without intervention.

**Clinical discussion:**

Femoral nerve palsy following epidural anesthesia is exceedingly rare and has not been previously documented. Differential diagnoses, including spinal hematoma and nerve compression, were excluded through comprehensive imaging. The transient nature of the palsy suggests a potential mechanical factor related to epidural catheter placement, such as catheter twisting or transient nerve irritation.

**Conclusion:**

This case underscores the importance of promptly evaluating new neurological deficits following epidural anesthesia in total hip arthroplasty patients. Early diagnosis and intervention are crucial to prevent lasting neurological damage, even in the absence of evident spinal lesions. Clinicians should consider mechanical factors related to catheter placement as potential causes of transient nerve palsy.

## Highlights

• Rare femoral nerve palsy after epidural in hip arthroplasty.
• Patient fully recovered within eight days post-surgery.
• MRI ruled out spinal compression or hematoma.
• Highlights importance of prompt evaluation of neurological deficits.

## 1 Introduction

Total hip replacement is a widely recognized and highly effective orthopedic procedure, with over one million surgeries performed globally each year [1]. Given its potential benefits and the limited risk of significant adverse effects, neuraxial anesthesia remains a frequently chosen option for both intraoperative anesthetic care as well as the provision of postoperative analgesia [[2], [3], [4]]. In addition to its analgesic benefits, which may exceed those of systemic opioid administration, epidural anesthesia effectively blunts the physiological and metabolic impact associated with tissue injury and surgical procedures [5]. This is especially true following lengthy and complex procedures which may result in activation of the sympathetic nervous system, leading to the release of inflammatory mediators including interlukin-6, interlukin-1, and tumor necrosis factor-alpha [5].

Spinal anesthesia, though generally safe, carries certain risks, including the rare possibility of permanent spinal cord or nerve damage, with an incidence of <4 cases per 100,000 [6,7]. Other potential complications include transient postoperative neurological deficits and post-puncture headaches [8].

Transient femoral nerve palsy following epidural catheterization, however, is exceedingly rare and has not been previously reported. In this case report, we describe a patient with femoral avascular necrosis and no prior neurological issues who developed right leg femoral nerve palsy following lumbar epidural catheter insertion for left hip total arthroplasty.

## 2 Case presentation

This work has been reported in line with the SCARE 2025 criteria [9].

A 44-year-old man, classified as ASA physical status I with a BMI of 24, diagnosed with femoral head avascular necrosis due to long-term use of prednisolone (5 mg daily for his asthma) underwent total hip arthroplasty (THA) in his left side. He had no history of surgical issues, an unremarkable family history, and denied any use of alcohol, tobacco, or illicit substances. Preoperative laboratory tests—including complete blood count, coagulation profile, electrolytes, and renal function—were within normal limits. The patient's perioperative care included standard monitoring with invasive blood pressure and central venous access, as well as the insertion of an 18G epidural catheter at the L2/3 level for postoperative pain management under local anesthesia.

The patient underwent a left cementless THA using an anterior approach while in the supine position, supported by a 10 cm pelvic bump following induction of general anesthesia. For induction, the patient received 2 mg of midazolam, 150 μg of fentanyl, 40 mg of atracurium, 40 mg of lidocaine, and 200 mg of propofol; anesthesia was maintained with 1.5 % enflurane throughout the procedure. The surgery lasted 145 min with an estimated blood loss of 350 cc. The surgery was uneventful, and the patient was successfully extubated shortly after the procedure, showing no signs of postoperative delirium or neurological issues. For postoperative pain management, a PCA pump delivered a continuous epidural infusion of 0.125 % ropivacaine with 1 μg/ml fentanyl at a rate of 4 ml/h. Postoperatively, the patient was prescribed ASA 80 mg twice daily, celecoxib 200 mg twice daily, and pantoprazole 40 mg daily. A thorough neurovascular examination of both lower extremities revealed normal findings.

Two days postoperatively, the patient presented with significant (M2) right quadriceps weakness accompanied by paresthesia affecting the anterior and medial thigh and medial leg, along with a diminished patellar reflex (while the Achilles reflex remained normal), which were not present prior to the anesthesia and surgery. Despite the epidural catheter being placed without incident, an urgent MRI of the thoracic, cervical and lumbar spine was conducted to rule out traumatic and non-traumatic causes of paresis. The MRI scans of the entire spine revealed no evidence of an epidural hematoma or other spinal lesions. The epidural catheter tip was correctly positioned in the L2 neuroforamina, but no stenosis or other abnormalities were detected at the site. Despite discontinuation of the PCA pump, the symptoms persisted.

On the morning of the third postoperative day, the epidural catheter was removed, and the patient was closely monitored. That evening, he reported a decrease in paresthesia, though motor strength remained unchanged. Paresthesia continued to improve and had completely resolved by the fifth day. Remarkably, the patient's motor condition began to improve five days after the operation, with strength in the right leg returning to M3. By the eighth postoperative day, the patient had fully recovered without any residual neurological deficits and did not require physical therapy during his hospital stay.

### 2.1 Imaging findings

#### 2.1.1 Pre-operative imaging

**Fig. 1:**
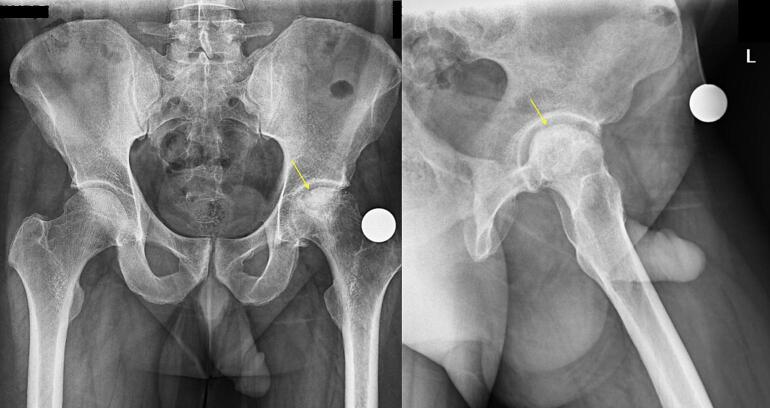
Pre-operative x-ray, left hip joint involvement. Left femoral head collapse along with osteonecrosis. A, AP view; B, Lateral view.

**Fig. 2:**
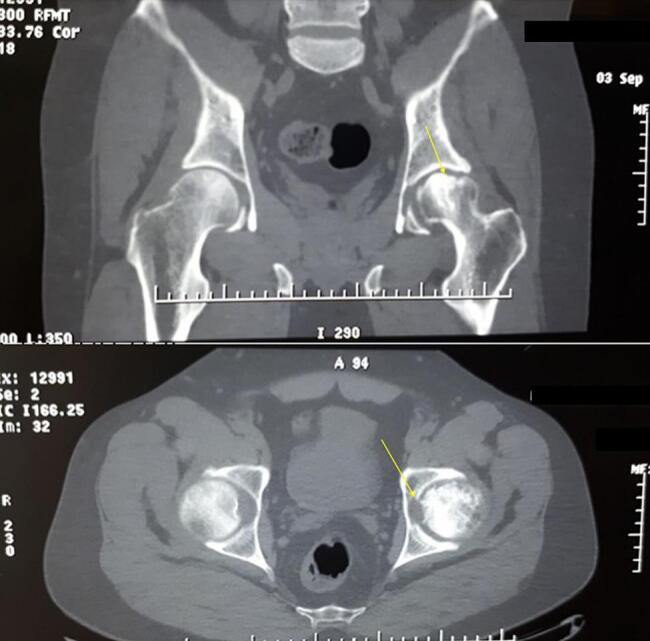
Axial (A) and coronal (B) view of pelvic CT scan. Left hip involved with femoral head collapse, subchondral hyper-density, osteoporosis, and osteonecrosis.

Diagnostic imaging included standard pelvic X-rays (anteroposterior and lateral views; Fig. 1) and a pelvic CT scan (Fig. 2). The imaging confirmed femoral head collapse, articular surface deformities, and osteoporosis, consistent with avascular necrosis.

#### 2.1.2 Post-operative imaging

**Fig. 3:**
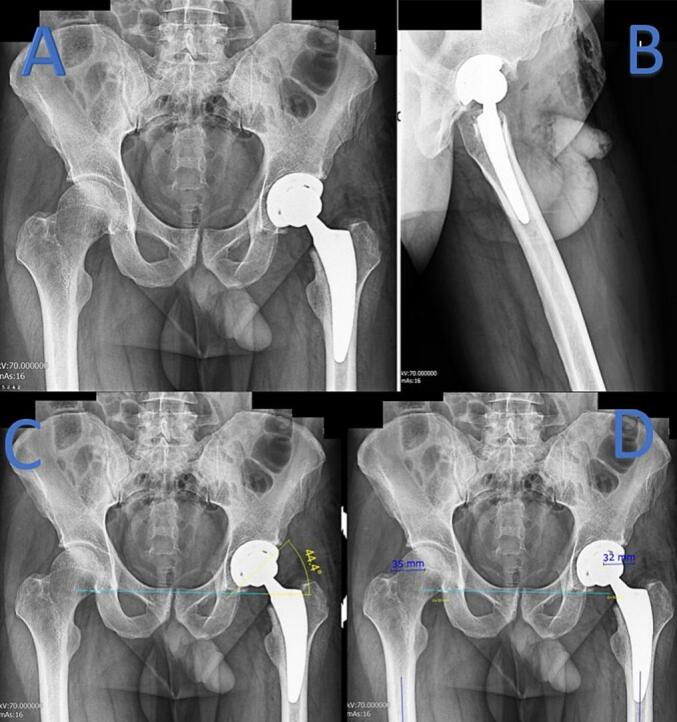
Post-operative pelvic x-ray. A: AP view, left hip successful prosthesis placement. B: Lateral view. C: Right (32 mm) and left (35) femoral offset. D: Left sharp angle (44.4°).

**Fig. 4:**
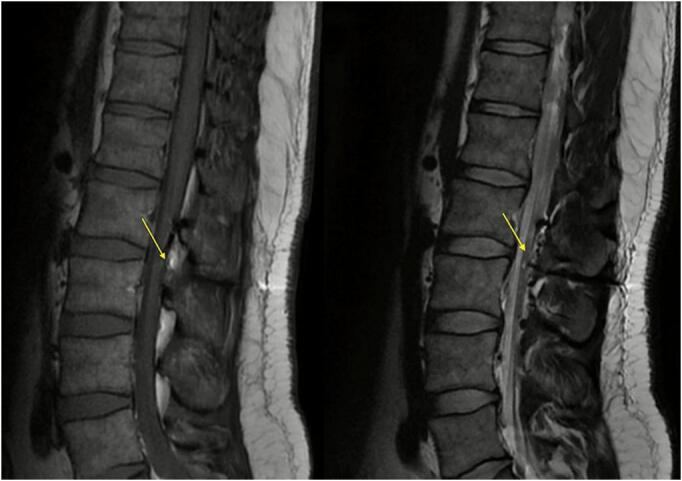
Sagittal T2W1 (A) and T1W1 (B) lumbar spine (L2−L3) MRI. Hemosiderin deposition representing a spontaneous resolved epidural hematoma.

Postoperative pelvic X-rays confirmed the successful implantation of the prosthesis, with an inclination angle of 44.4° and a femoral offset of 35 mm in the left hip (Fig. 3). The right hip femoral offset measured 32 mm. A postoperative spinal MRI, performed due to the patient's right femoral nerve palsy, showed no evidence of an epidural hematoma, disc herniation and stenosis at the L2 neuroforamina where the epidural catheter was placed (Fig. 4). Hemosiderin deposition at the catheter tip was observed after catheter removal 2 days post-surgery, following the onset of symptoms and performed MRI, likely indicative of a resolved previous hematoma.

## 3 Discussion

While serious complications from epidural anesthesia are rare, it is crucial to promptly evaluate any new neurological symptoms following the placement of an epidural catheter to prevent potential neurological damage [5,[10], [11], [12]]. Oliveira et al. identified possible diagnoses, such as stroke, myasthenia gravis, severe hypokalemia, and spinal cord compression, that can be ruled out by an urgent imaging test, such as a CT or MRI scan, or blood gas analysis [13].

Although our case is, to our knowledge, the first to describe an isolated femoral nerve palsy after anterior-approach total hip arthroplasty with a correctly sited thoraco-lumbar epidural, the broader literature shows that transient lower-limb motor deficits after epidural catheterisation are uncommon but not unprecedented [[14], [15], [16], [17], [18]]. Collectively, these reports share three themes: (i) neurological deficits often appear within 6–72 h of catheter insertion, (ii) neuro-imaging is frequently negative, and (iii) clinical recovery is typically complete within days to months—paralleling the trajectory seen in our patient.

The femoral nerve arises from the L2–L4 roots within the psoas compartment before passing beneath the inguinal ligament. Although the epidural space is anatomically dorsal to the exiting nerve roots, tangential needle or catheter advancement can breach the paravertebral gutter and contact the lumbar plexus. Cadaveric dye-distribution studies demonstrate that an overshoot of only 5–10 mm beyond the posterior ligamentum flavum may reach the dural sleeve or ventral epidural tissues where the plexus resides [19,20]. Iatrogenic mechanical trauma typically produces an immediate, dermatomally restricted motor deficit that does not progress, mirroring the clinical picture we observed.

Acute femoral nerve palsy can arise anywhere along the L2-L4 root-to-nerve continuum, and the differential spans both central and peripheral compression. Central causes include disc prolapse or foraminal stenosis and, more dramatically, intraspinal or paravertebral haematoma—lesions that may escape early MRI detection when <2 mm in size  [21]. Peripheral entrapment is most often reported inside the psoas/iliacus compartment, where even a small retro- or intramuscular haematoma can stretch the nerve against the iliacus fascia and produce quadriceps weakness and groin paraesthesia  [22]. Outside the pelvis, direct mechanical factors dominate: mal-positioned anterior acetabular retractors, excessive leg-lengthening, or prolonged hip extension/external rotation in the supine anterior-THA set-up can kink or tension the nerve beneath the inguinal ligament, mimicking neuraxial injury  [23,24]. Comparable neuropraxias have also been described after lithotomy or obstetric stirrups, strengthening the link between lower-limb posture and transient femoral conduction block  [25].

Chronic glucocorticoid therapy does not directly injure peripheral nerves, but it does create a milieu that can predispose to transient femoral palsy. Long-term steroids accelerate type-II-fibre atrophy and weaken peri-neural soft tissue, lowering the threshold for stretch-induced neurapraxia during forceful hip positioning  [26]. They also reduce capillary integrity and, when combined with antiplatelet or anticoagulant agents typical of postoperative regimens, markedly increase the risk of spontaneous or minimal-trauma iliopsoas/retroperitoneal haematoma—the single most common non-surgical cause of postoperative femoral neuropathy  [21].

Spinal epidural hematoma is the most feared explanation for acute neurologic decline after neuraxial procedures. MRI, however, may miss hematomas <2 mm or collections located in the paravertebral muscles and psoas sheath that compress the lumbar plexus rather than the cord [27,28]. Risk factors include multiple needle passes, coagulopathy, and periprocedural anticoagulation. Our patient had none of these, and serial MRI performed at 6 h and 48 h was negative, but a micro-hematoma causing transient plexus ischemia cannot be fully excluded.

Once the Tuohy needle is correctly sited, even midline catheters can deviate laterally and form loops or knots. Fluoroscopic studies reveal that up to 20 % of thoraco-lumbar catheters cross the midline, and 5 % exit through an intervertebral foramen [29,30]. Such migration may exert a mass-like effect on the adjacent nerve root or plexus, or impair perfusion via compression of the radicular vessels. The rapid onset and equally rapid recovery in our patient are consistent with a reversible neurapraxia caused by intermittent kinking or torsion of the catheter tip.

All local anesthetics are potentially neurotoxic at sufficient concentration and exposure time. Experimental data show that bupivacaine ≥0.5 % can trigger mitochondrial dysfunction and apoptosis in sensory and motor neurons [[31], [32], [33]]. Additives such as epinephrine or dexamethasone may potentiate this effect by reducing regional blood flow. In vivo, neurotoxicity usually manifests as a delayed deficit with patchy distribution, yet isolated femoral involvement has been reported, particularly when catheter portals lie preferentially toward one side. The low-dose ropivacaine regimen (0.2 %) used in our case makes this explanation less likely but not impossible.

In a study by Vetter and colleagues, a case was reported where a patient developed transient tetraparesis after epidural anesthesia for a Whipple operation [34]. The patient had no neurological symptoms preoperatively, but an emergency MRI was performed immediately after the operation to rule out a cervical cord lesion. MRI showed complete spinal canal stenosis in the vasculature of the right sulco-commissural artery at C4/5 level, probably caused by the patient's position during the 6-h operation. The likelihood of transient hypotension leading to chronic myelon damage was considered unlikely.

In another 2014 study, Doctor et al. described a patient with renal cell carcinoma who had metastases in the lumbar vertebrae [35]. The patient became paraplegic after insertion of a thoracic epidural catheter for nephrectomy. The patient had metastases in the thoracic epidural space that had not been previously diagnosed. When the epidural catheter was removed, these metastases likely bled and caused compressive myelopathy, resulting in irreversible paraplegia.

In our case, despite thorough imaging and clinical evaluation, there were no signs of bone metastasis, hematoma, abscess, or other lumbar lesions that could explain the patient's neurological symptoms. The only plausible explanation our team identified was a possible twisting of the epidural catheter within the relevant space, leading to transient neurological impairment.

## 4 Conclusion

This case highlights the importance of immediate and thorough evaluation of new neurological symptoms following epidural anesthesia, even when the initial placement appears uneventful. Although serious complications are rare, they can lead to significant morbidity if not promptly addressed. Our case underscores the need for clinicians to consider all potential causes, including mechanical factors such as catheter positioning, when faced with unexplained neurological deficits. Early intervention and appropriate imaging are crucial to ruling out serious conditions and ensuring patient safety and recovery.

## Informed consent

The patient gave informed consent for publication.

## Ethical approval

This work is a single-patient case report and does not involve any experimental intervention or use of identifiable private information beyond standard clinical practice. According to the policies and regulations of IKHC, TUMS, a separate ethical approval is not required for case reports. The patient's anonymity has been maintained, and written informed consent was obtained for the publication of this report.

## Funding

No funding was received for this work.

## Author contribution

MP, MR, PBS, MG, MAJ, SMJM contribute to writing of this paper. This article is supervised by SMJM.

## Guarantor

SMJM is the Guarantor of this work.

## Research registration number

N/A.

## Declaration of Generative AI and AI-assisted technologies in the writing process

Yes

AI was used solely for grammar and language editing during the manuscript writing and revision stages.

No generative AI was used for developing research questions, analyzing data, creating figures, or drafting scientific content.

The authors take full responsibility for the integrity and accuracy of the manuscript content.

The tool used was ChatGPT (OpenAI, GPT-4, June 2024 version).

Usage occurred between May and June 2025 via the ChatGPT web interface (cloud-based).

No custom plug-ins or fine-tuning were used. Default parameters were applied.

Only non-sensitive manuscript text was input for grammar editing.

No patient data, clinical images, or identifiable information were shared.

All usage was compliant with GDPR/HIPAA regulations. No data-sharing agreements were required.

The manuscript was reviewed entirely by the corresponding and senior authors.

All AI-edited content was carefully fact-checked and revised by the authors.

No AI-generated scientific content was used. Only edited grammar was retained.

We acknowledge the limitations of AI tools and confirm human oversight at all stages.

Since AI use was limited to grammar editing, bias and algorithmic influence on scientific content were not applicable.

No financial or professional ties to AI vendors exist.

Ethical use of AI tools was maintained throughout.

Because AI was only used for grammar editing, no scientific artefacts were generated.

No code, prompts, or supplementary materials are necessary for replication.

## Conflict of interest statement

No conflicts of interest to declare.
